# Effect of Cardiac Myosin Inhibitors on Echocardiographic Features of Cardiac Structure and Function in Hypertrophic Cardiomyopathy: A Systematic Review and Meta-Analysis

**DOI:** 10.31083/RCM45043

**Published:** 2026-01-20

**Authors:** Yang Lu, Yuanyuan Zhu, Zhuang Tian

**Affiliations:** ^1^Department of Cardiology, State Key Laboratory of Complex Severe and Rare Diseases, Peking Union Medical College Hospital, Chinese Academy of Medical Sciences & Peking Union Medical College, 100730 Beijing, China; ^2^International Medical Service, Peking Union Medical College Hospital, Chinese Academy of Medical Sciences and Peking Union Medical College, 100730 Beijing, China

**Keywords:** hypertrophic cardiomyopathy, mavacamten, aficamten, cardiac myosin inhibitor, echocardiographic, meta-analysis

## Abstract

**Background:**

Recent advancements have introduced novel cardiac myosin inhibitors (CMIs) that have demonstrated significant efficacy in treating hypertrophic cardiomyopathy (HCM). This meta-analysis aimed to clarify the current understanding of the impact of CMIs on echocardiographic cardiac structure and function in patients with HCM.

**Methods:**

A comprehensive search of the PubMed, Cochrane Library, and Embase databases was conducted from inception until September 14, 2025. The studies reporting the impact of CMIs on echocardiographic cardiac structure and function in HCM patients were included.

**Results:**

Ultimately, this meta-analysis included 10 studies: five randomized controlled trials (RCTs), three echocardiographic sub-studies derived from RCTs, and two long-term cohort studies. A total of 938 patients were enrolled in these studies. This meta-analysis revealed that CMIs significantly reduce interventricular septum thickness (mean difference (MD): –1.77, 95% confidence interval (CI): –3.30 to –0.23; *p* = 0.0240). CMIs were also shown to significantly reduce left ventricular mass index (MD: –18.15, 95% CI: –32.65 to –3.65; *p* = 0.0141). Moreover, the pooled results demonstrated that administering CMIs can significantly reduce left ventricular ejection fraction (MD: –3.22, 95% CI: –5.60 to –0.85; *p* = 0.0078). CMIs also significantly improved echocardiographic parameters of left ventricular diastolic function, such as the left atrial volume index (MD: –5.75, 95% CI: –7.87 to –3.64; *p* < 0.0001) and septal E/e′ ratio (MD: –3.80, 95% CI: –4.74 to –2.87; *p* < 0.0001). However, the results did not reveal an association between CMIs and the risk of atrial arrhythmias (risk ratio (RR): 0.98, 95% CI: 0.33 to 2.94; *p* = 0.9689).

**Conclusions:**

CMIs have shown great efficacy in improving left ventricular structure and diastolic function in HCM patients. Additionally, CMIs can reduce left ventricular ejection fraction. However, the impact of CMIs on the risk of atrial arrhythmias remains unclear.

**The PROSPERO Registration:**

CRD420251243904, https://www.crd.york.ac.uk/PROSPERO/view/CRD420251243904.

## 1. Introduction

Hypertrophic cardiomyopathy (HCM) is a common genetic disorder marked by the abnormal thickening of the left ventricular wall. This condition frequently results in severe complications including heart failure, arrhythmias, and sudden cardiac death [1]. HCM is categorized into obstructive HCM (oHCM) and nonobstructive HCM (nHCM) based on the left ventricular outflow tract obstruction (LVOTO) [1]. Traditional management strategies have focused on symptom alleviation through nonspecific therapies. Recent advances in treatment have introduced cardiac myosin inhibitors (CMIs), such as mavacamten and aficamten, which target sarcomeric dysfunction at the molecular level, offering a more tailored therapeutic approach [2, 3]. Clinical trials have demonstrated that these agents significantly improved LVOTO, symptoms, and heart failure biomarkers in patients with both oHCM and nHCM [4, 5, 6, 7, 8].

Echocardiography serves as the principal imaging technique for the majority of patients. Essential insights derived from echocardiography include the establishment of diagnosis and the assessment of associated structural and functional cardiac abnormalities. Characterization of dynamic LVOTO is a key strength of echocardiography. The recording of maximal wall thickness, systolic function and LV apical aneurysms all contribute to the assessment of prognosis [1].

Improvements in LVOTO caused by mavacamten and aficamten have been adequately described in their respective randomized controlled trials (RCTs). Additionally, meta-analyses [9, 10] have explored the effects of CMIs on LVOTO, New York Heart Association (NYHA) class, and heart failure biomarkers. However, there is currently no meta-analysis or systematic review specifically addressing changes in echocardiographic parameters among HCM patients after CMIs treatment. Therefore, this analysis aimed to summarize the effects of CMIs on echocardiographic features in HCM patients. Patients with HCM frequently experience atrial arrhythmias, including atrial fibrillation (AF) and atrial flutter (AFL). One study indicated that the prevalence of AF in HCM patients is approximately 20% to 25% [11]. The mechanisms underlying the occurrence of atrial arrhythmias in HCM are linked to left ventricular hypertrophy and structural remodelling of the left atrium. Therefore, we also discussed the impact of CMIs on HCM patients with concurrent atrial arrhythmias.

## 2. Materials and Methods

This meta-analysis was performed in accordance with the guidelines outlined in the Preferred Reporting Items for Systematic Reviews and Meta-analyses as well as the Cochrane Handbook [12, 13].

### 2.1 Search Strategy

A comprehensive search was conducted in PubMed, Cochrane Library and Embase databases by using the keywords: “hypertrophic cardiomyopathy”, “HCM”, “mavacamten”, “aficamten”, “cardiac myosin inhibitor”, “CMI”, “echocardiography”, “echocardiogram”, and “echocardiographic” for studies available up to September 14, 2025. The comprehensive search strategy for each database is available in the **Supplementary Materials**. Subsequently, the eligibility criteria were applied for screening.

### 2.2 Eligibility Criteria and Study Selection

We included human studies in this study if they met the following inclusion criteria: (1) participants must be adults aged 18 years and older, with a confirmed diagnosis of hypertrophic cardiomyopathy; (2) outcomes must include echocardiographic measures of cardiac structure and function, as well as any reported adverse effects; (3) studies must report outcomes via standardized echocardiographic techniques and measurements; and (4) no language restrictions will be applied, but only studies with full-text availability will be included. The exclusion criteria were as follows: (1) case reports or reviews; (2) studies that did not report relevant echocardiographic measures; and (3) studies that did not provide sufficient data for meta-analysis or lacked clarity in methodology.

### 2.3 Data Extraction

Data were systematically gathered by two authors into a spreadsheet designated for analytical purposes. Any inconsistencies were addressed through collaborative discussion. A third researcher was involved to resolve any discrepancies. The information retrieved from the qualifying studies comprised the study titles, dates of publication, sample sizes, baseline characteristics of participants, types of treatment administered, duration of the treatment, and baseline echocardiographic features. We extracted data from studies on arrhythmia events, including AF and AFL. We also extracted the mean difference (MD) and 95% confidence interval (CI) or standard deviation in the change from baseline (CFB) of echocardiographic parameters between the treatment and placebo groups. These parameters include interventricular septum thickness (IVST) or maximal left ventricular wall thickness (MLVWT), left ventricular mass index (LVMI), left ventricular ejection fraction (LVEF), left atrial volume index (LAVI), lateral e^′^velocity, septal e^′^ velocity, lateral E/e^′^ ratio, and septal E/e^′^ ratio. For the single-arm cohort study, which aimed to observe the long-term efficacy of CMIs without a control group, we extracted the CFB for the above echocardiographic parameters.

### 2.4 Quality of Assessment

Two authors independently evaluated the risk of bias in the included studies using the Cochrane Risk of Bias Tool (RoB2) [14] for RCTs and the Newcastle-Ottawa scale (NOS) [15] for the cohort study. A third researcher was involved to resolve any discrepancies.

### 2.5 Statistical Analysis

This meta-analysis was conducted using the “meta” package in R software version 4.4.2 (R Foundation for Statistical Computing, Vienna, Austria; https://www.r-project.org/). The results are expressed as risk ratio (RR) and MD along with a 95% CI. Heterogeneity among the included studies was evaluated using the *I*^2^ statistic, where an *I*^2^ value exceeding 50% suggests the presence of at least moderate heterogeneity. Given the premise of considerable clinical heterogeneity, a random-effects model was utilized for this meta-analysis. For RCTs, we chose the MD in echocardiographic parameters within the CFB between the treatment and placebo groups as the measure of treatment effect. For single-arm cohort studies, we used the CFB of echocardiographic parameters for the meta-analysis. For effects with substantial heterogeneity, we conducted sensitivity and subgroup analyses to explore the sources of heterogeneity. Since most methods of detecting publication bias are only applicable to more than 10 studies included, we have only created the funnel plots.

## 3. Results

### 3.1 Study Selection

**Fig. 1.**
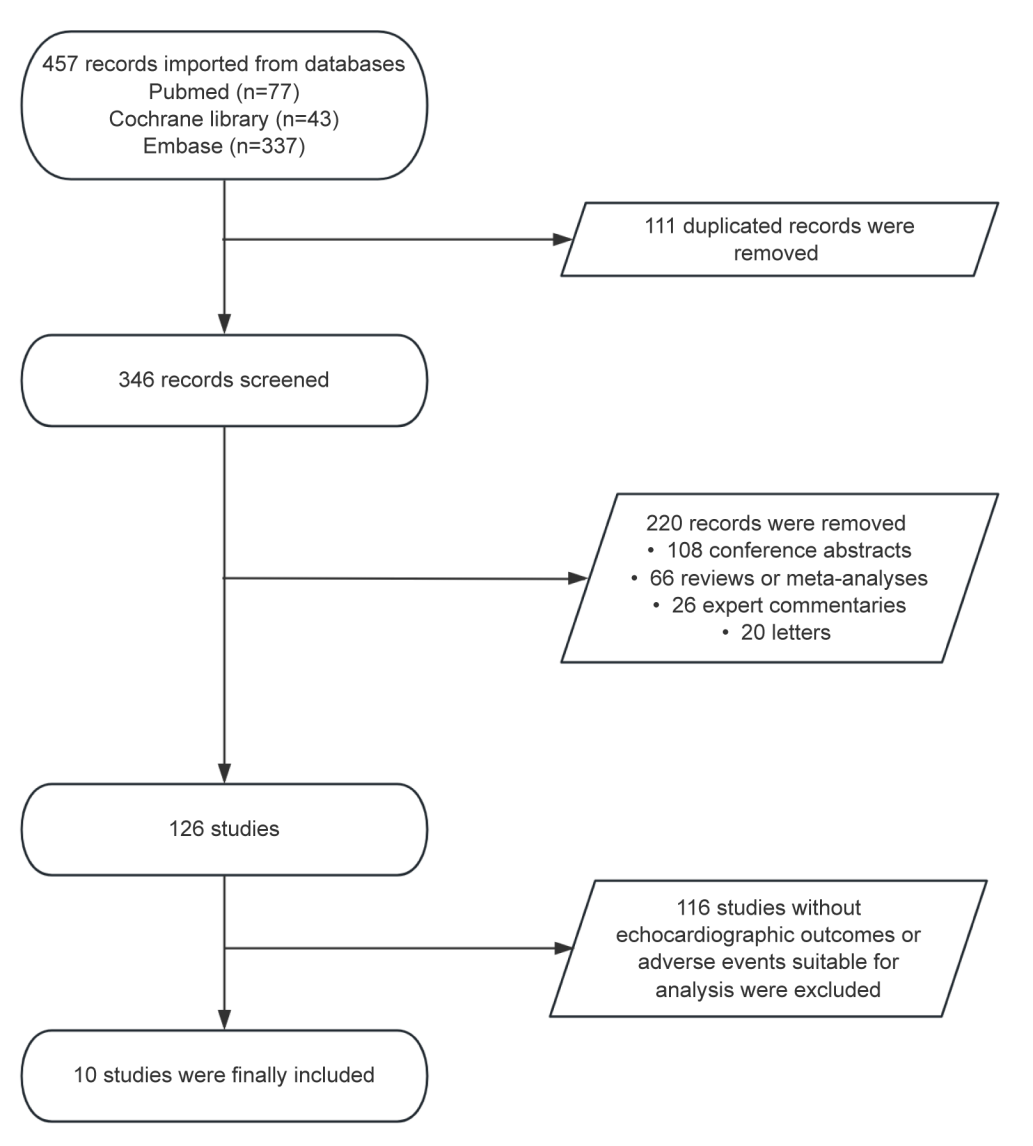
**Preferred reporting items for systematic review and meta-analysis (PRISMA) flowchart of the study screen**.

A total of 457 records were obtained from Pubmed, Cochrane Library and Embase databases. Among them, 111 records were deleted because of duplication, and 220 records were excluded on the basis of titles and abstracts. Among them, there are 108 conference abstracts, 66 reviews or meta-analyses, 26 expert commentaries, and 20 letters. Next, 116 studies that did not report echocardiographic outcomes or adverse events suitable for analysis were excluded. Finally, 10 studies [4, 5, 6, 7, 8, 16, 17, 18, 19, 20] met the eligibility criteria (Fig. 1). These 10 studies include 1 phase II RCT (MAVERICK-HCM [4]), 4 phase III RCTs (EXPL-ORER-HCM [5], EXPLORER-CN [6], SEQUOIA-HCM [7] and VALOR-HCM [8]), their corresponding 3 substudies of echocardiographic features [16, 17, 18], and 2 long-term cohort studies [19, 20].

### 3.2 Study Characteristics

Table 1 (Ref. [4, 5, 6, 7, 8, 16, 17, 18, 19, 20]) summarizes the main characteristics of the ten studies. A total of 938 patients were enrolled in these studies, with the majority being male. The studies reported a mean patient age between 51.0 and 60.9 years. With the exception of Ho *et al*., 2020 [4], who enrolled patients with nHCM, all other studies focused on patients with oHCM. Olivotto *et al*., 2020 [5], Hegde *et al*., 2021 [16], Tian *et al*., 2023 [6] and Tian *et al*., 2025 [17] administered mavacamten with dose titration guided by echocardiographic measurements of LVEF and LVOTO, with a treatment duration of 30 weeks. Maron *et al*., 2024 [7] and Hegde *et al*., 2024 [18] used aficamten, with dose adjustments based on LVEF and LVOTO, and a treatment period of 24 weeks. Desai *et al*., 2022 [8] was a phase 3 RCT named the VALOR-HCM trial, which enrolled oHCM patients who had indications for septal reduction therapy (SRT). Desai *et al*., 2022 [8] was designed with a relatively short primary endpoint timeline of 16 weeks, which may be particularly relevant for patients considering SRT [21]. Desai *et al*., 2025 [19] is a long-term follow-up study of the VALOR-HCM trial. After 16 weeks, patients in the placebo group crossed over to receive mavacamten treatment and were followed up to 128 weeks, with corresponding data collected. Saberi *et al*., 2025 [20] is also a long-term cohort study, which enrolled 46 patients and continued aficamten treatment for up to 48 weeks. Table 2 (Ref. [8, 16, 17, 18, 19, 20]) presents the echocardiographic characteristics of the included studies. All patients exhibited ventricular wall thickness and LVMI above the normal upper limits, indicating severe LV structural abnormalities in oHCM patients. Elevated LAVI and E/e^′^ ratios suggest significant LV diastolic dysfunction in this population. LVEF ranged from 67.9% to 77.8%, slightly higher than in healthy individuals, indicating a compensatory increase in LV systolic function.

**Table 1.** **Main characteristics of all the studies included in the meta-analysis**.

Study	RCT name	Diagnosis	No. (treatment/placebo)	Sex, male/female	Age (years), treatment vs. placebo	Treatment	Treatment duration
Ho *et al*., 2020 [4]	MAVERICK-HCM	nHCM	58 (39/19)	24/34	54.0 ± 14.6 vs. 53.8 ± 18.2	Mavacamten	24 weeks
Olivotto *et al*., 2020 [5] and Hegde *et al*., 2021 [16]	EXPLORER-HCM	oHCM	251 (123/128)	149/102	58.5 ± 12.2 vs. 58.5 ± 11.8	Mavacamten	30 weeks
Tian *et al*., 2023 [6] and Tian *et al*., 2025 [17]	EXPLORER-CN	oHCM	81 (54/27)	58/23	52.4 ± 12.1 vs. 51.0 ± 11.8	Mavacamten	30 weeks
Maron *et al*., 2024 [7] and Hegde *et al*., 2024 [18]	SEQUOIA-HCM	oHCM	282 (142/140)	167/115	59.2 ± 12.6 vs. 59.0 ± 13.3	Aficamten	24 weeks
Desai *et al*., 2022 [8]	VALOR-HCM: Week 16 Results	oHCM	112 (56/56)	57/55	59.8 ± 14.2 vs. 60.9 ± 10.5	Mavacamten	16 weeks
Desai *et al*., 2025 [19]	VALOR-HCM: Week 128 Results	oHCM	108	54/54	60.3 ± 12.5	Mavacamten	128 weeks
Saberi *et al*., 2025 [20]	FOREST-HCM: 48-Week Results	oHCM	46	20/26	59.7 ± 12.8	Aficamten	48 weeks

nHCM, nonobstructive hypertrophic cardiomyopathy; oHCM, obstructive hypertrophic cardiomyopathy; RCT, randomized controlled trial.

**Table 2.** **Baseline echocardiographic features of the studies included in the meta-analysis**.

Study	RCT name	MLVWT (mm)*	LVMI (g/m^2^)*	LVEF (%)*	LAVI (mL/m^2^)*	Lateral e^′^ (cm/s)*	Septal e^′^ (cm/s)*	Lateral E/e^′^*	Septal E/e^′^*
Hegde *et al*., 2021 [16]	EXPLORER-HCM	17.0 ± 3.0/17.0 ± 3.0	112.0 ± 27.0/110.0 ± 26.0	74.0 ± 6.0/74.0 ± 6.0	40.0 ± 12.0/41.0 ± 14.0	6.0 ± 2.0/7.0 ± 2.0	5.0 ± 1.0/5.0 ± 2.0	15.0 ± 6.0/15.0 ± 8.0	20.0 ± 7.0/20.0 ± 9.0
Tian *et al*., 2025 [17]	EXPLORER-CN	22.9 ± 4.9/24.3 ± 6.4	152.3 ± 47.8/174.3 ± 73.2	77.8 ± 6.9/77.0 ± 6.7	43.3 ± 12.1/47.5 ± 14.7	6.6 ± 2.0/5.5 ± 2.6	4.5 ± 1.3/4.1 ± 1.6	14.0 ± 7.5/16.8 ± 6.7	19.8 ± 7.6/20.8 ± 4.9
Hegde *et al*., 2024 [18]	SEQUOIA-HCM	20.7 ± 3.0/21.0 ± 3.0	129.6 ± 31.0/134.6 ± 36.6	75.0 ± 6.0/75.0 ± 6.0	40.1 ± 12.7/40.9 ± 15.1	6.0 ± 2.0/6.1 ± 2.2	4.6 ± 1.4/4.6 ± 1.6	15.4 ± 7.3/15.9 ± 7.8	19.5 ± 8.4/20.5 ± 9.3
Desai *et al*., 2022 [8]	VALOR-HCM: Week 16 Results	18.0 ± 4.0/17.0 ± 4.0	119.2 ± 29.5/122.3 ± 30.7	67.9 ± 3.7/68.3 ± 3.2	41.3 ± 16.5/40.9 ± 15.2	6.3 ± 2.2/7.1 ± 1.8	4.9 ± 1.5/5.3 ± 1.5	15.5 ± 7.4/13.5 ± 5.7	19.6 ± 9.1/18.1 ± 7.6
Desai *et al*., 2025 [19]	VALOR-HCM: Week 128 Results	21.0 ± 3.0	119.2 ± 29.5	68.2 ± 3.3	40.9 ± 15.9	-	-	-	19.6 ± 9.1
Saberi *et al*., 2025 [20]	FOREST-HCM: 48-Week Results	19.6 ± 3.1	129.4 ± 26.9	69.0 ± 5.0	36.0 ± 8.0	-	-	-	18.9 ± 7.7

*For the RCTs, all baseline echocardiographic characteristics are presented separately for the treatment and placebo groups. For the single-arm studies, only the baseline echocardiographic characteristics of the overall population are shown. LAVI, left atrial volume index; LVEF, left ventricular ejection fraction; LVMI, left ventricular mass index; MLVWT, maximal left ventricular wall thickness.

### 3.3 Risk of Bias

Table 3 (Ref. [4, 5, 6, 7, 8, 16, 17, 18]) and Table 4 (Ref. [19, 20]) present the risk of bias of the studies [4, 5, 6, 7, 8, 16, 17, 18, 19, 20] included in this meta-analysis. All of included RCTs [4, 5, 6, 7, 8, 16, 17, 18] were judged as having a low risk of bias.

**Table 3.** **The risk of bias assessment in included RCTs**.

Study	Bias arising from the randomization process	Bias due to deviations from the intended intervention	Bias due to missing outcome data	Bias in measurement of the outcome	Bias in selection of the reported results	Other risk of bias	Overall judgement
Ho *et al*., 2020 [4]	Low	Low	Low	Low	Moderate	Low	Low
Olivotto *et al*., 2020 [5] and Hegde *et al*., 2021 [16]	Low	Low	Low	Low	Low	Low	Low
Tian *et al*., 2023 [6] and Tian *et al*., 2025 [17]	Low	Low	Low	Low	Low	Low	Low
Maron *et al*., 2024 [7] and Hegde *et al*., 2024 [18]	Low	Low	Low	Low	Low	Low	Low
Desai *et al*., 2022 [8]	Low	Low	Low	Low	Low	Low	Low

**Table 4.** **The risk of bias assessment in the included cohort studies**.

Study	Selection	Comparability	Exposure	Quality scores
Desai *et al*., 2025 [19]	****	**	***	9
Saberi *et al*., 2025 [20]	****	**	***	9

The risk of bias for cohort studies was assessed using the Newcastle–Ottawa Scale (NOS). This scale consists of three domains: selection, comparability, and exposure, each containing corresponding evaluation items. * is used to indicate awarded points. The maximum scores are 4* for selection, 2* for comparability, and 3* for exposure, with a total maximum score of 9*. Higher scores indicate higher study quality.

### 3.4 LV Structure

**Fig. 2.**
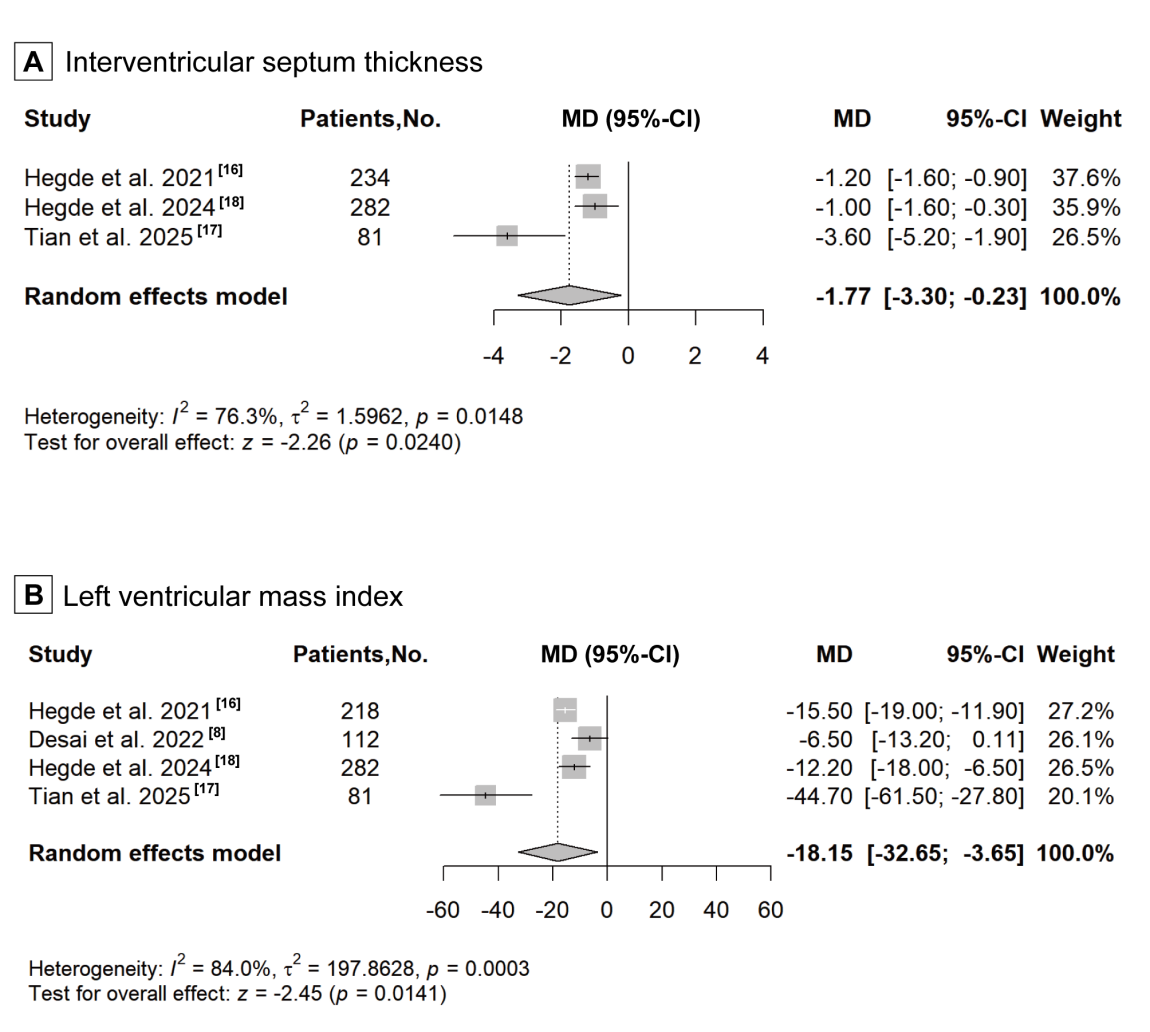
**Forest plot for improvement of left ventricular structure, including two echocardiographic parameters: (A) interventricular septum thickness, and (B) left ventricular mass index**. CI, confidence interval; MD, mean difference.

Hegde *et al*., 2021 [16], Hegde *et al*., 2024 [18] and Tian *et al*., 2025 [17] reported changes in IVST, whereas Hegde *et al*., 2021 [16], Desai *et al*., 2022 [8], Hegde *et al*., 2024 [18] and Tian *et al*., 2025 [17] reported changes in LVMI to reflect structural remodelling of the left ventricle after treatment. The pooled analysis reveals that CMIs significantly decreased IVST levels compared with those in the placebo group (MD: –1.77, 95% CI: –3.30 to –0.23, *p* = 0.0240; Fig. 2A). The aggregated results reveals that CMIs also significantly reduced the LVMI (MD: –18.15, 95% CI: –32.65 to –3.65, *p* = 0.0141; Fig. 2B). The two single-arm cohort studies by Desai *et al*., 2025 [19] and Saberi *et al*., 2025 [20] both reported changes in LV wall thickness and LVMI. Analysis results show that CMIs significantly decreased MLVWT compared to baseline (MD: –1.16, 95% CI: –1.51 to –0.81, *p* < 0.0001; **Supplementary Fig. 1A**). The findings also suggest that CMIs reduced LVMI (MD: –12.05, 95% CI: –16.46 to –7.63, *p* < 0.0001; **Supplementary Fig. 1B**). **Supplementary Fig. 2** presents the sensitivity analysis for IVST. It was found that heterogeneity significantly decreased when Tian *et al*., 2025 [17] was excluded, whereas heterogeneity remained high when either of the other two studies was removed. **Supplementary Fig. 3** shows the subgroup analysis for LVMI. Four studies were divided into two groups based on sample size. The results indicate that the subgroup with larger sample sizes exhibited lower heterogeneity. Since the treatment duration of CMIs in Desai *et al*., 2022 [8] was shorter than that in other RCTs, a subgroup analysis was conducted to explore the impact of different treatment durations on the efficacy of CMIs. **Supplementary Fig. 4A** shows that there is no significant difference in the improvement of LVMI between subgroups with different treatment durations (*p* = 0.1170).

### 3.5 LV Systolic Function

**Fig. 3.**
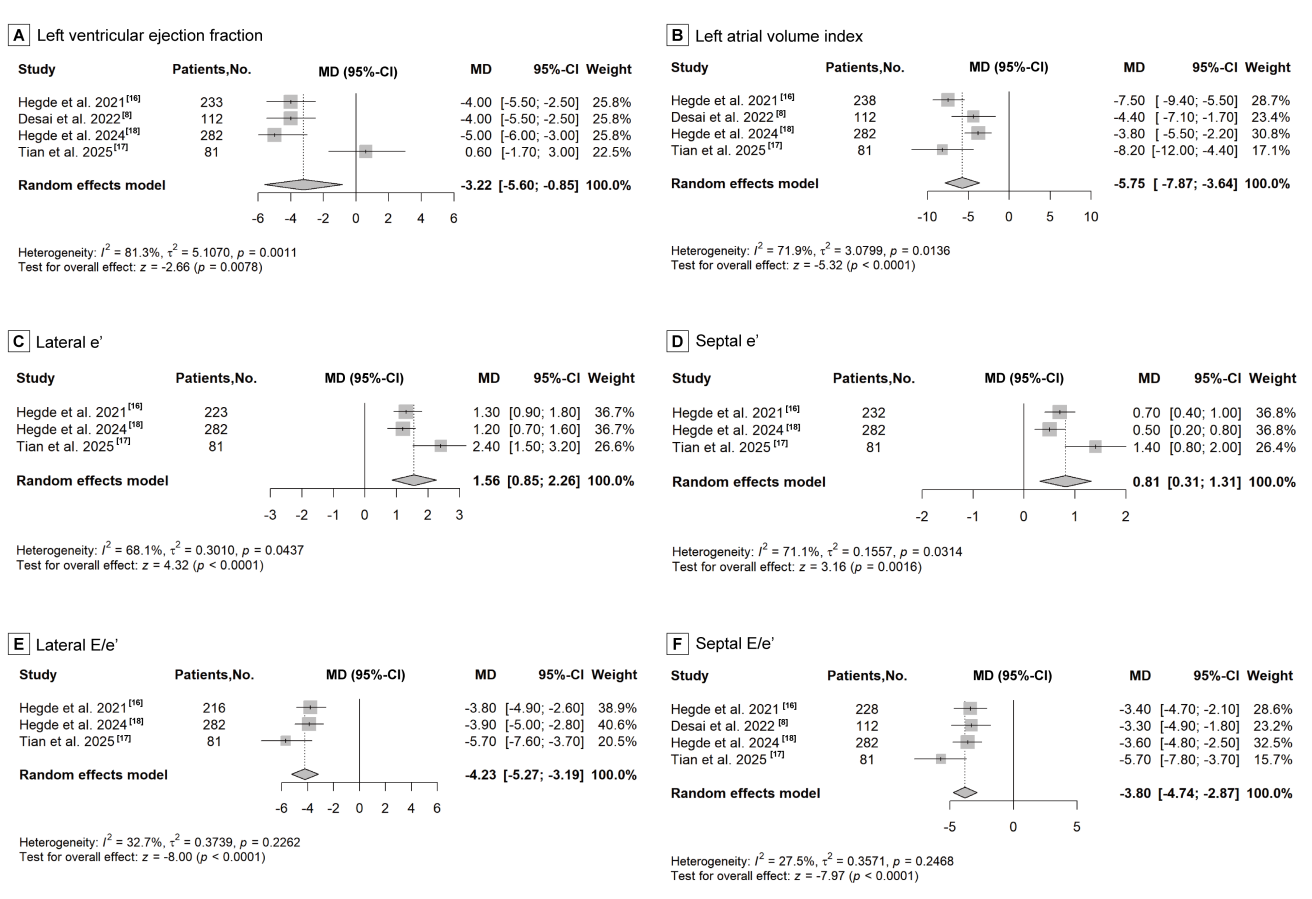
**Forest plot for improvement of left ventricular function, including six echocardiographic parameters: (A) left ventricular ejection fraction, (B) left atrial volume index, (C) lateral e^′^ velocity, (D) septal e^′^ velocity, (E) lateral E/e^′^ ratio, and (F) septal E/e^′^ ratio**.

LVEF is the echocardiographic parameter reflecting LV systolic function and was reported in Hegde *et al*., 2021 [16], Desai *et al*., 2022 [8], Hegde *et al*., 2024 [18] and Tian *et al*., 2025 [17]. However, the reported changes in LVEF across the four studies [8, 16, 18, 20] were inconsistent. Compared with those in the placebo group, Hegde *et al*., 2021 [16], Desai *et al*., 2022 [8] and Hegde *et al*., 2024 [18] reported a statistically significant reduction in LVEF after treatment with CMIs. Tian *et al*., 2025 [17] reported a slight, nonsignificant increase in LVEF compared with placebo. The pooled analysis demonstrates an overall statistically significant reduction in LVEF (MD: –3.22, 95% CI: –5.60 to –0.85, *p* = 0.0078; Fig. 3A). The combined results of the two long-term cohort studies also demonstrate a decrease in LVEF following treatment (MD: –4.88, 95% CI: –5.96 to –3.79, *p* < 0.0001; **Supplementary Fig. 1C**). **Supplementary Fig. 4B** shows that treatment of different duration results in a decrease in LVEF, with no significant difference between groups (*p* = 0.5530).

### 3.6 LV Diastolic Function

Hegde *et al*., 2021 [16], Hegde *et al*., 2024 [18] and Tian *et al*., 2025 [17] reported five echocardiographic parameters reflecting LV diastolic function, including LAVI, lateral e^′^, septal e^′^, lateral E/e^′^, and septal E/e^′^. Desai *et al*., 2022 [8] reported only LAVI and septal E/e^′^. Significant improvement in LAVI (MD: –5.75, 95% CI: –7.87 to –3.64, *p* < 0.0001; Fig. 3B), lateral e^′^ (MD: 1.56, 95% CI: 0.85 to 2.26, *p* < 0.0001; Fig. 3C), septal e^′^ (MD: 0.81, 95% CI: 0.31 to 1.31, *p* = 0.0016; Fig. 3D), lateral E/e^′^ (MD: –4.23, 95% CI: –5.27 to –3.19, *p* < 0.0001; Fig. 3E), and septal E/e^′^ (MD: –3.80, 95% CI: –4.74 to –2.87, *p* < 0.0001; Fig. 3F) were observed in the aggregated results. The combined results of the two long-term cohort studies also demonstrate improvement in LAVI (MD: –3.65, 95% CI: –5.20 to –2.10, *p* < 0.0001; **Supplementary Fig. 1D**) and septal E/e^′^ (MD: –3.75, 95% CI: –6.00 to –1.50, *p* = 0.0011; **Supplementary Fig. 1E**). **Supplementary Fig. 4C,D** demonstrate that diastolic function improves across subgroups with different treatment duration, with no significant differences between them (*p* > 0.05).

### 3.7 Atrial Arrhythmias

**Fig. 4.**
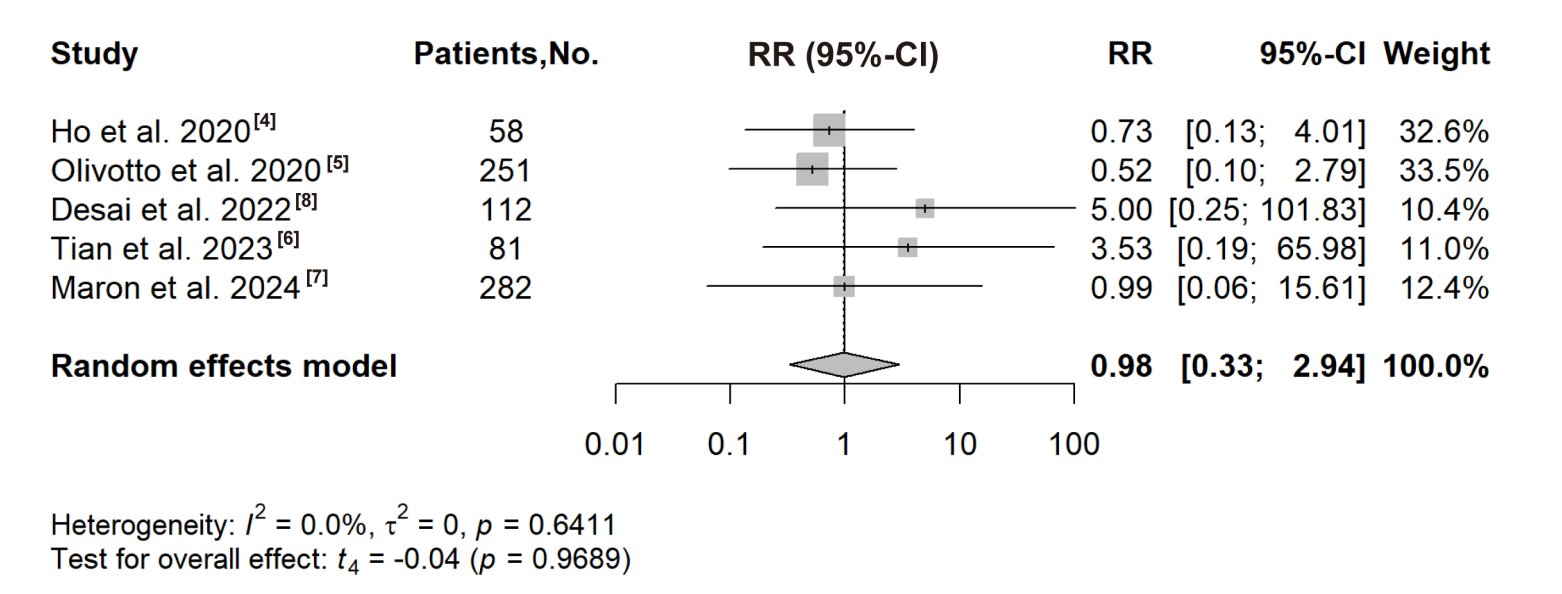
**Forest plot for the risk of atrial arrhythmias**. RR, risk ratio.

Ho *et al*., 2020 [4], Olivotto *et al*., 2020 [5], Desai *et al*., 2022 [8], Tian *et al*., 2023 [6] and Maron *et al*., 2024 [7] reported the number of atrial arrhythmia events in the treatment group and the placebo group. The results of these five RCTs did not show that receiving CMIs treatment increased the risk of atrial arrhythmias. No significant association with the risk of atrial arrhythmias was observed in the meta-analysis (RR: 0.98, 95% CI: 0.33 to 2.94, *p* = 0.9689; Fig. 4).

## 4. Discussion

The results of this meta-analysis provided evidence regarding the effects of CMIs on LV structure and function in patients with oHCM. The main findings are as follows: (1) CMIs treatment significantly improved LV structure of patients with HCM, as evidenced by reduction in IVST and LVMI; (2) CMIs significantly increased the likelihood of reduced LVEF; (3) CMIs treatment led to notable improvement in the LV diastolic function of HCM patients; and (4) there is no clear conclusion regarding whether CMIs increased the risk of atrial arrhythmias.

HCM is an inherited cardiomyopathy characterized by myocardial hypertrophy that is typically caused by genetic mutations. Abnormal interactions between actin and myosin within cardiomyocytes result in excessive myocardial contractility even in the absence of significant afterload, leading to dynamic obstruction. This phenomenon occurs in approximately 75% of patients with HCM [22]. The primary impact of HCM is diastolic dysfunction of the LV. Myocardial hypertrophy hinders effective relaxation of the LV, leading to elevated filling pressure and subsequent symptoms of congestive heart failure [23]. The emergence of CMIs has offered hope for improving clinical outcomes of HCM patients. Currently approved CMIs include mavacamten and aficamten, which selectively inhibit cardiac-specific myosin activity to reduce myocardial contractility. These agents modulate the interaction between actin and myosin, thereby reducing contractile force in cardiomyocytes, alleviating symptoms, and improving cardiac function [24]. With the advent of CMIs, the value of echocardiography has further increased. It serves not only as a baseline assessment tool but also as an essential modality for monitoring treatment efficacy [25]. With the publication of studies about echocardiographic features after CMIs treatment [4, 5, 6, 7, 8, 16, 17, 18, 19, 20], we performed an updated systematic review and meta-analysis to provide higher-level evidence for clinical decision-making.

Hegde *et al*., 2021 [16], Desai *et al*., 2022 [8], Hegde *et al*., 2024 [18] and Tian *et al*., 2025 [17] consistently reported significant reductions in IVST and LVMI among oHCM patients, which was further supported by our meta-analysis. The results for these two parameters indicate substantial heterogeneity. Further sensitivity analysis shows that heterogeneity significantly decreased after excluding the study with a small sample size. Subgroup analysis also demonstrates lower heterogeneity in the subgroup with larger sample sizes. These findings suggest that a small sample size may be a major source of the observed heterogeneity.

As CMIs suppress myocardial contractility, close monitoring of LVEF during treatment is essential. However, findings on LVEF changes varied among the four studies [8, 16, 18, 20]. This variability may be attributed to the lower initial dose of mavacamten (2.5 mg daily) used by Tian *et al*., 2023 [6], which likely had a smaller effect on LVEF. Despite this, this meta-analysis indicated that CMIs significantly reduce LVEF, underscoring the need for careful LVEF monitoring during dose titration. At the same time, this also indicates that some advanced oHCM patients with already reduced LVEF are unable to undergo CMIs. Additionally, for oHCM patients who have undergone septal myectomy, the reduced myocardial mass may make it difficult to tolerate the side effects associated with further LVEF decline. The study by Tian *et al*. (2023) [6] showed that changes in LVEF before and after treatment were not statistically significant, which further suggests that a lower starting dose may be more appropriate for patients experiencing rapid LVEF reduction. Multiple RCT protocols have emphasized rigorous LVEF monitoring for all patients, with discontinuation of CMIs if LVEF declines too rapidly. CMIs should be initiated only when LVEF is above 55%. Dose escalation is not recommended if LVEF falls below 55%. If LVEF decreases to less than 50%, CMIs should be discontinued. Echocardiography should continue to be performed every 4 weeks, and treatment can be restarted at a reduced dose once LVEF recovers to more than 50% [5, 6, 7]. Therefore, no heart failure events related to CMI-induced LVEF reduction have been observed to date. In contrast to systolic function, both studies [8, 16, 17, 18, 19, 20] and our meta-analysis have consistently shown that CMIs significantly improve LV diastolic function in HCM patients. As HCM commonly presents as heart failure with preserved ejection fraction (HFpEF) [26], our findings further support that CMIs improve diastolic function by reducing LVOTO and improving LV structure, thereby lowering filling pressures and alleviating HFpEF symptoms. In addition to improving LVOTO and reversing LV hypertrophy, CMIs can also enhance cardiac biomarkers such as N-terminal pro-B-type natriuretic peptide and cardiac troponins. This observation suggests that CMIs may improve left ventricular diastolic function by reducing ventricular wall stress and mitigating myocardial cell injury.

In this meta-analysis, the treatment duration of CMIs in Desai *et al*., 2022 [8] was shorter than that in the other RCTs. However, the subgroup analysis reveals no significant differences between groups. This may be because the 16-week treatment period in Desai *et al*., 2022 [8] was still sufficient for mavacamten to exert its therapeutic effects. Nonetheless, a greater disparity in treatment duration could potentially lead to differences in the efficacy of CMIs.

In HCM patients, atrial structural remodelling often manifests as left atrial (LA) enlargement and morphological changes. This remodelling results from pressure overload due to LV hypertrophy and LV diastolic function. LA enlargement alters electrophysiological properties, promoting abnormal excitability and reduced conduction velocity in cardiomyocytes, which in turn increases the risk of atrial arrhythmias, particularly AF [27]. Since CMIs reduce LV hypertrophy and filling pressure, they theoretically have the potential to decrease the incidence of atrial arrhythmias. Nonetheless, both Desai *et al*., 2022 [8] and Tian *et al*., 2023 [6] observed a higher incidence of atrial arrhythmia occurrences within the mavacamten cohort compared to the placebo cohort, although these differences were not statistically significant. Similarly, our meta-analysis revealed no statistically significant association between CMIs and the incidence of atrial arrhythmia. This may be due to the relatively short follow-up durations in the included studies [4, 5, 6, 7, 8], which may not adequately capture new-onset atrial arrhythmias. Additionally, the treatment durations in these studies may have been insufficient to observe the potential arrhythmia-reducing effects of CMIs. Interestingly, a substudy [28] from the VALOR-HCM trial focusing on LA function reported significant improvements in LA conduit strain, contraction strain, and reservoir strain after 56 weeks of mavacamten treatment. The improvement in atrial strain also physiologically suggests that the incidence of atrial arrhythmias may not necessarily increase. The increased risk of atrial arrhythmias reported in the studies by Desai *et al*., 2022 [8] and Tian *et al*., 2023 [6] may suggest that short-term data could underestimate this risk, highlighting the need for long-term follow-up. Therefore, the effect of CMIs on atrial arrhythmias warrants further investigation in larger-scale studies with longer follow-up periods.

### Limitations

Our meta-analysis has several limitations. First, although this meta-analysis included a total of 10 studies, the primary results regarding echocardiographic characteristics were derived mainly from 4 RCTs. The small number of eligible RCTs precluded a methodologically rigorous evaluation of publication bias using quantitative tests (e.g., Egger’s test), which typically require ≥10 studies to achieve sufficient statistical power. While funnel plots (**Supplementary Fig. 5**) were generated for visual inspection, their interpretability remains constrained under such limited conditions. Furthermore, the included studies presented a short mean follow-up period. Therefore, additional studies with extended follow-up durations are essential to verify the long-term effects. Since this meta-analysis only included RCTs involving oHCM, its conclusions cannot be applied to nHCM. More randomized controlled trials are needed in the future to validate the effects of CMIs in nHCM. Additionally, this meta-analysis combined the effects of two types of CMIs on oHCM without differentiating between their individual impacts. Existing literature has indicated that CMIs can improve mitral regurgitation (MR) in patients with oHCM. However, the studies included in our meta-analysis did not report on changes in MR. Therefore, this meta-analysis is unable to assess the impact of CMIs on MR. This meta-analysis specifically focused on the effects of CMIs on echocardiographic characteristics in HCM patients and did not address cardiac magnetic resonance or cardiac biomarker.

## 5. Conclusions

This meta-analysis confirms that, in the short term, mavacamten and aficamten can improve the LV structure and diastolic function in HCM patients, as evidenced by improvements in the IVST, LVMI, LAVI, e^′^ and E/e^′^ as measured by echocardiography. CMIs can also lead to a decrease in LVEF. Therefore, close monitoring of LVEF and timely adjustment of CMIs therapy are essential. However, the impact of CMIs on the risk of atrial arrhythmias remains unclear.

## Availability of Data and Materials

Not applicable.

## Supplementary Material

Supplementary material associated with this article can be found, in the online version, at https://doi.org/10.31083/RCM45043.
