# Supplementary material for: Effect of Cardiac Myosin Inhibitors on Echocardiographic Features of Cardiac Structure and Function in Hypertrophic Cardiomyopathy: A Systematic Review and Meta-Analysis
Source: Rev Cardiovasc Med. 2026 Jan 20;27(1):45043. doi: 10.31083/RCM45043 (PMC12873658; doi:10.31083/RCM45043)
Supplement: Supplementary file 1 [file 2153-8174-27-1-45043-s1.zip › Supplementary Materials.docx]

**Supplementary Materials**

**Detailed search strategy**

**Pubmed：**

#1 Hypertrophic cardiomyopathy[Title/Abstract] OR HCM[Title/Abstract]

#2 Mavacamten[Title/Abstract] OR Aficamten[Title/Abstract] OR Cardiac myosin inhibitor[Title/Abstract] OR CMI[Title/Abstract]

#3 Echocardiography[Title/Abstract] OR Echocardiogram[Title/Abstract] OR Echocardiographic[Title/Abstract]

#4 #1 AND #2 AND #3

**Cochrane library:**

#1 (Hypertrophic cardiomyopathy):ti,ab,kw OR (HCM):ti,ab,kw

#2 (Mavacamten):ti,ab,kw OR (Aficamten):ti,ab,kw OR (Cardiac myosin inhibitor):ti,ab,kw OR (CMI):ti,ab,kw

#3 (Echocardiography):ti,ab,kw OR (Echocardiogram):ti,ab,kw OR (Echocardiographic):ti,ab,kw

#4 #1 AND #2 AND #3

**Embase：**

#1 'Hypertrophic cardiomyopathy' OR 'HCM'

#2 'Mavacamten' OR 'Aficamten' OR 'Cardiac myosin inhibitor' OR 'CMI'

#3 'Echocardiography' OR 'Echocardiogram' OR 'Echocardiographic'

#4 #1 AND #2 AND #3

**
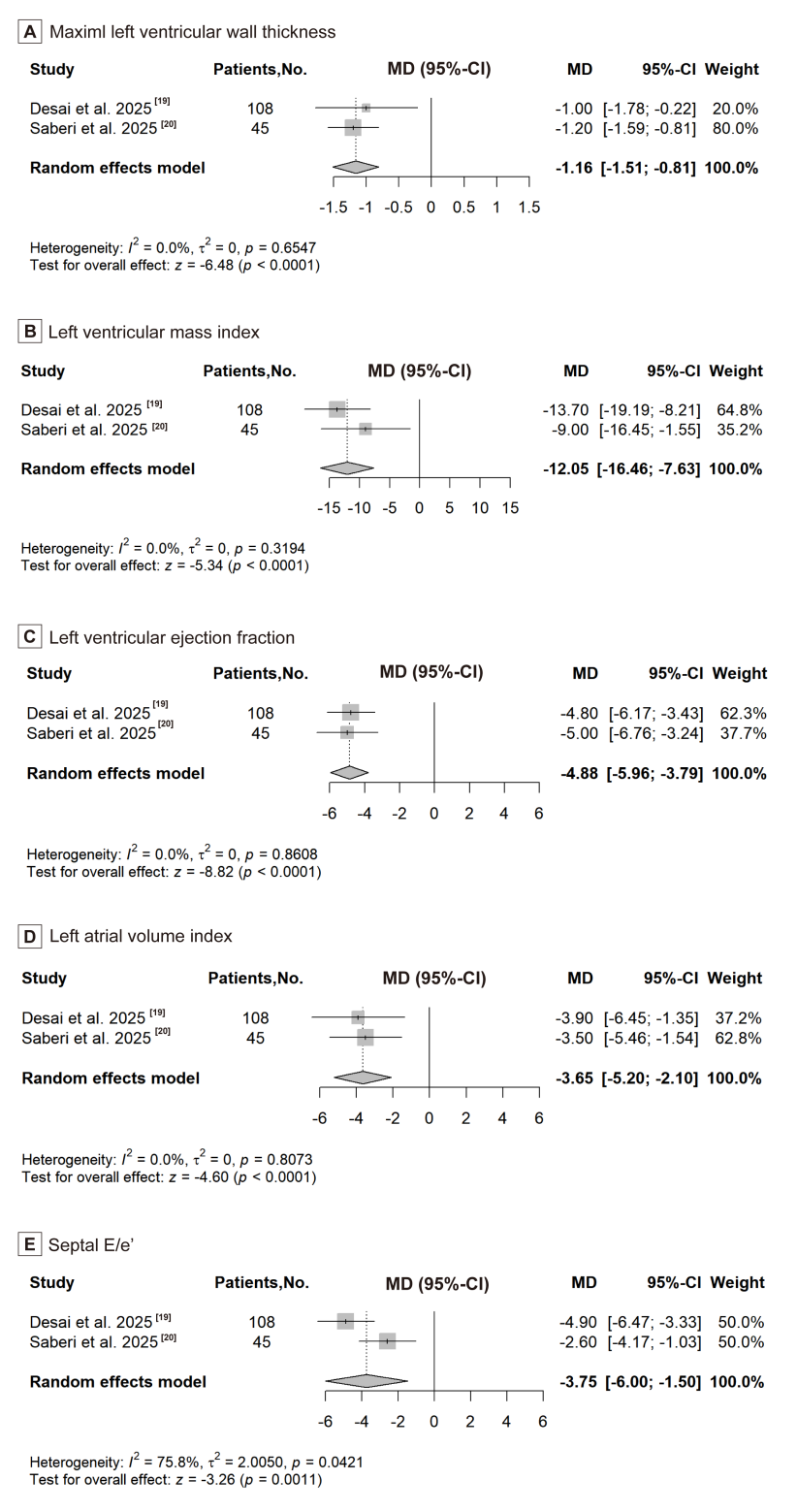
Supplementary Fig. 1.** Forest plot for improvement of left ventricular structure and function (results from Desai et al. 2025 [19] and Saberi et al. 2025 [20]), including five echocardiographic parameters: (A) maximal left ventricular wall thickness, (B) left ventricular mass index, (C) left ventricular ejection fraction, (D) left atrial volume index, (E) septal E/e' ratio. CI, confidence interval; MD, mean difference.

**
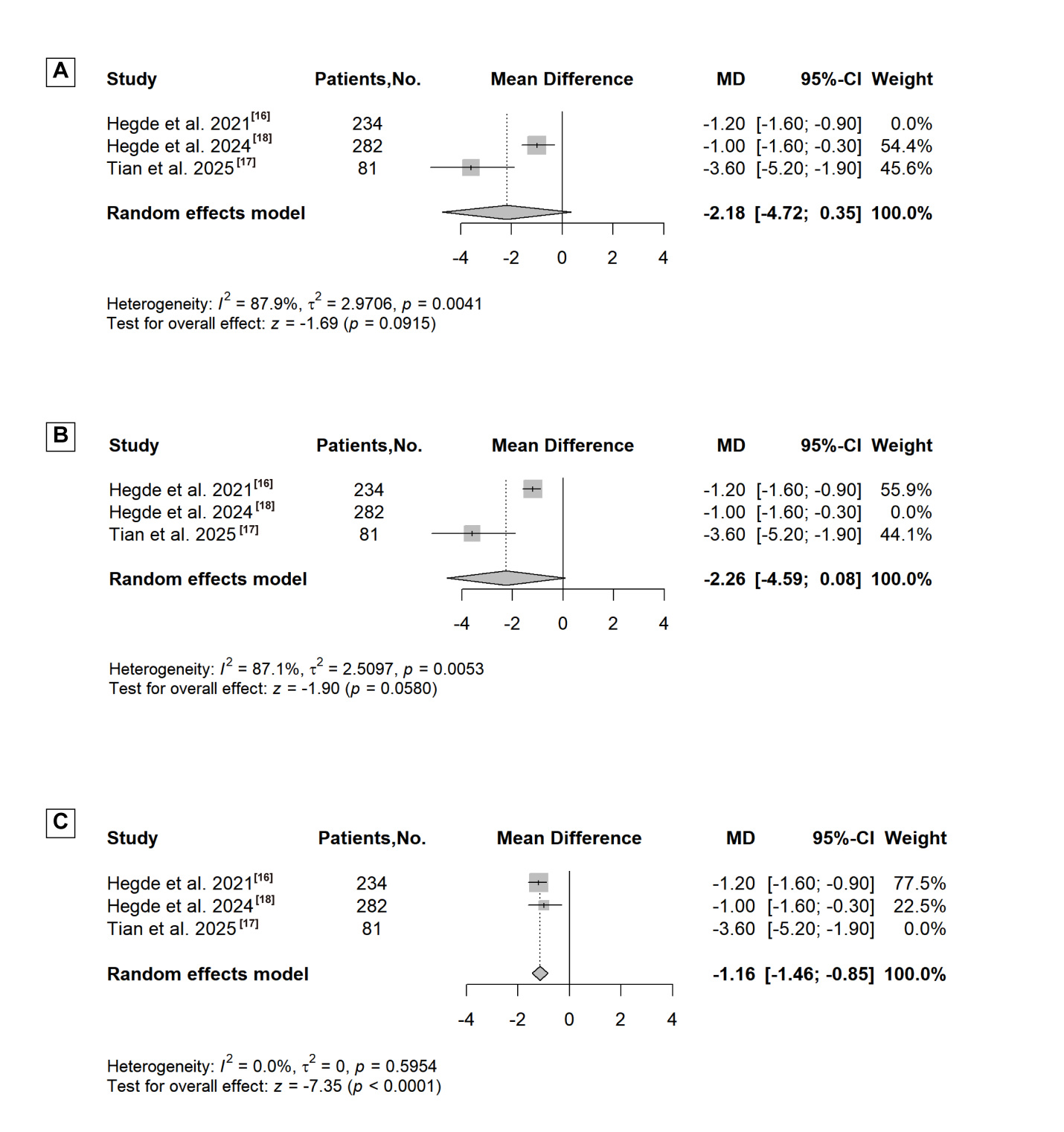
Supplementary Fig. 2.** Sensitivity analysis of the effects of CMIs on interventricular septal thickness in oHCM patients. (A) results after excluding Hegde et al. 2021 [16]; (B) results after excluding Hegde et al. 2024 [18]; (C) results after excluding Tian et al. 2025 [17]. CI, confidence interval; CMIs, cardiac myosin inhibitors; MD, mean difference; oHCM, obstructive hypertrophic cardiomyopathy.


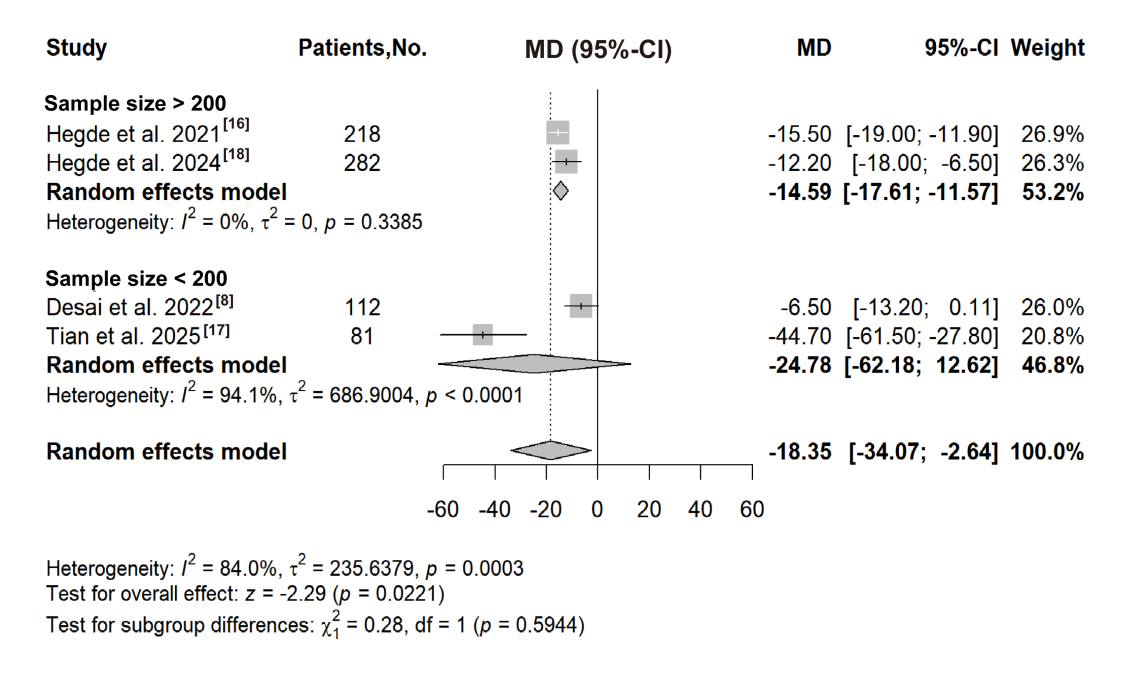
**Supplementary Fig. 3.** Subgroup analysis of the effects of CMIs on left ventricular mass index improvement in oHCM patients based on sample size. CI, confidence interval; CMIs, cardiac myosin inhibitors; MD, mean difference; oHCM, obstructive hypertrophic cardiomyopathy.


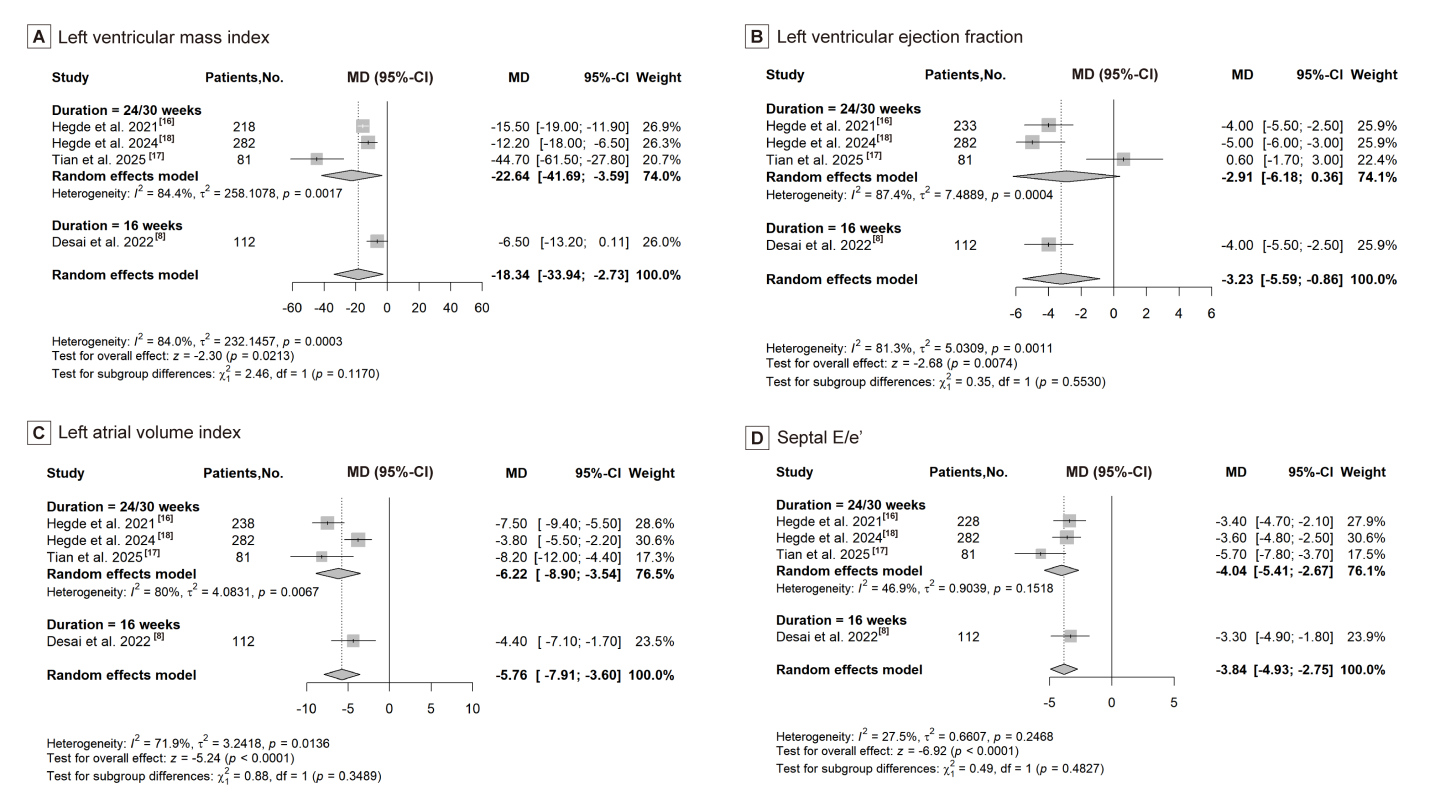


**Supplementary Fig. 4.** Subgroup analysis based on treatment duration for the effects of CMIs on four echocardiographic parameters: (A) left ventricular mass index; (B) left ventricular ejection fraction; (C) left atrial volume index; (D) septal E/e’. CI, confidence interval; CMIs, cardiac myosin inhibitors; MD, mean difference; oHCM, obstructive hypertrophic cardiomyopathy.

**
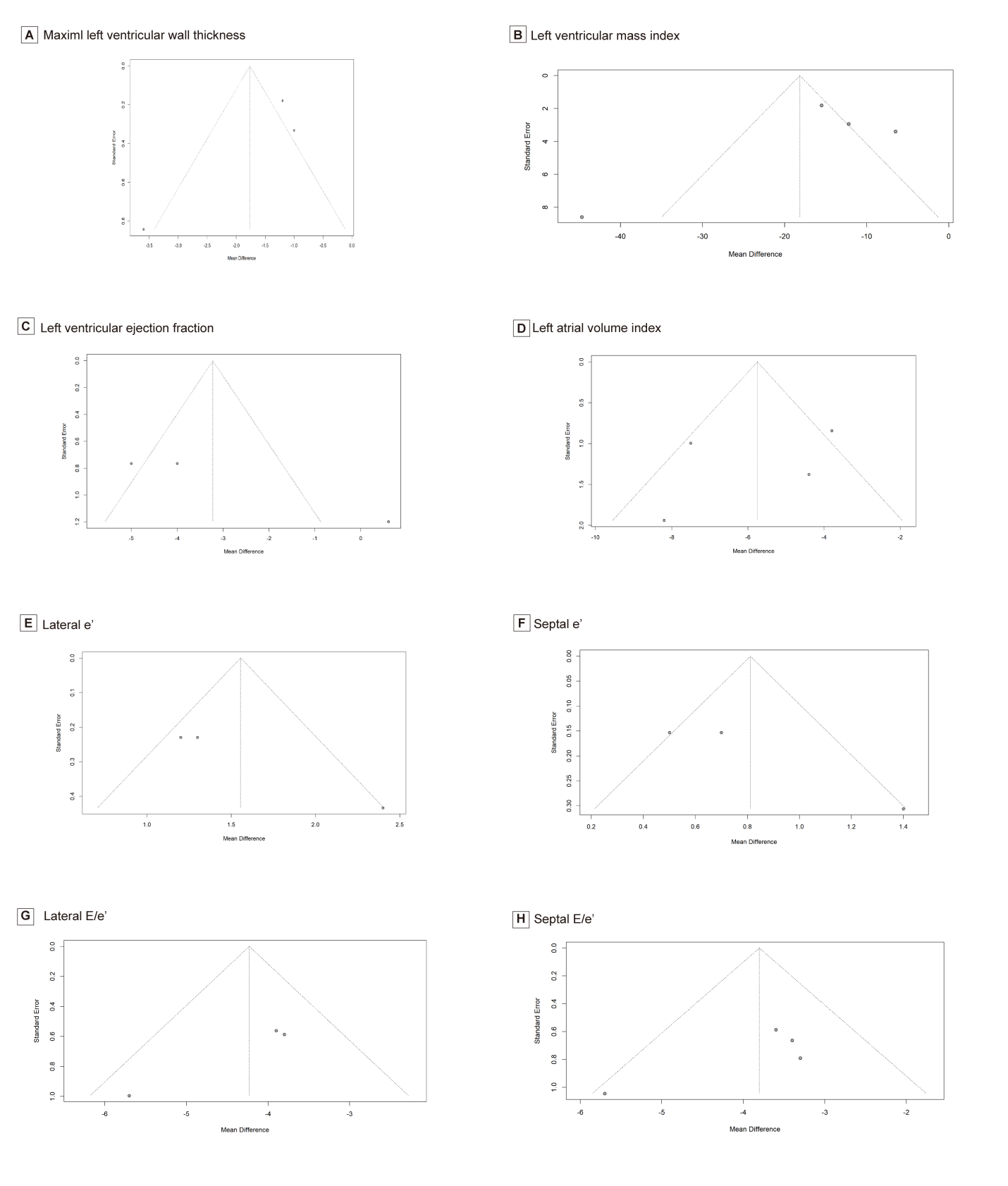
Supplementary Fig. 5**. Funnel plots for eight echocardiographic parameters: (A) maximal left ventricular wall thickness, (B) left ventricular mass index, (C) left ventricular ejection fraction, (D) left atrial volume index, (E) lateral e' velocity, (F) septal e' velocity, (G) lateral E/e' ratio, and (H) septal E/e' ratio.
